# Neuromorphic log-domain silicon synapse circuits obey bernoulli dynamics: a unifying tutorial analysis

**DOI:** 10.3389/fnins.2014.00428

**Published:** 2015-01-20

**Authors:** Konstantinos I. Papadimitriou, Shih-Chii Liu, Giacomo Indiveri, Emmanuel M. Drakakis

**Affiliations:** ^1^Bioinspired VLSI Circuits and Systems Group, Department of Bioengineering, Imperial College LondonLondon, UK; ^2^Institute of Neuroinformatics, University of Zurich and ETH ZurichZurich, Switzerland

**Keywords:** analog VLSI (aVLSI), generalized bernoulli cell formalism, log-domain circuits, subthreshold MOSFETs, synaptic dynamics

## Abstract

The field of neuromorphic silicon synapse circuits is revisited and a parsimonious mathematical framework able to describe the dynamics of this class of log-domain circuits in the aggregate and in a systematic manner is proposed. Starting from the Bernoulli Cell Formalism (BCF), originally formulated for the modular synthesis and analysis of externally linear, time-invariant logarithmic filters, and by means of the identification of new types of Bernoulli Cell (BC) operators presented here, a generalized formalism (GBCF) is established. The expanded formalism covers two new possible and practical combinations of a MOS transistor (MOST) and a linear capacitor. The corresponding mathematical relations codifying each case are presented and discussed through the tutorial treatment of three well-known transistor-level examples of log-domain neuromorphic silicon synapses. The proposed mathematical tool unifies past analysis approaches of the same circuits under a common theoretical framework. The speed advantage of the proposed mathematical framework as an analysis tool is also demonstrated by a compelling comparative circuit analysis example of high order, where the GBCF and another well-known log-domain circuit analysis method are used for the determination of the input-output transfer function of the high (4^th^) order topology.

## 1. Introduction

Almost 20 years ago, a novel, systematic, transistor-level formalism for the analysis and synthesis of externally-linear, internally-nonlinear (ELIN) (Tsividis, 1997) log-domain filters was introduced. The formalism was termed “*The Bernoulli Cell Formalism*” in an attempt to highlight its key element, the Bernoulli differential equation describing the time-dependent behavior of a forward-biased BJT collector current when a linear capacitor is connected to its emitter terminal (Drakakis et al., 1997b). A similar mathematical description holds for the drain current of a weakly-inverted MOST, when a linear capacitor is connected to its source terminal.

By arranging many BC topologies in cascade form, where the input of the next BC becomes the output of the previous one, a set of general coupled equations termed “*Log-Domain State Space*” (LDSS) is generated (Drakakis et al., 1999b). The resulting set of linearised differential equations of the LDSS stems from the non-linear differential equation governing each BC and constitutes a powerful and handy tool, well suited for the transfer function derivation of any order of log-domain filter. Several high-order log-domain filter circuit examples in literature confirm the above statement and identify the BCF as a parsimonious analysis and synthesis tool (Drakakis et al., 1997a; Drakakis, 2006; Ip et al., 2009; Katsiamis et al., 2009; Kardoulaki et al., 2013).

A review of past literature reveals that the BCF constitutes a complete, systematic mathematical framework not only for ELIN but also for intrinsically non-linear log-domain circuits. When it comes to the synthesis of purely non-linear log-domain circuits, a variant of the BCF, termed Non-linear Bernoulli Cell Formalism (NBCF), is able to implement challenging non-linear dynamics, based on the “*Coupled BC Formation*,” where the input and output currents of the BCs are interconnected in a non-sequential way, in contrast to the cascaded LDSS topology. A new category of bioinspired circuits, termed “*CytoMimetic*” has thus been born, which is able to emulate cellular and molecular dynamics in a systematic manner (Papadimitriou and Drakakis, 2012; Papadimitriou et al., 2013). Intriguingly, apart from the aforementioned linear and non-linear VLSI systems, Bernoulli dynamics are identified in the case of ideal memristors as well (Drakakis and Payne, 2000a; Drakakis et al., 2010; Georgiou et al., 2012a,b). The resemblance between the dynamics of ideal memristors and artificial or not synaptic circuits has been identified repeatedly in literature.

From the research so far, one could claim that the BCF is a “*chimera*” formalism, able to describe both linear and non-linear state-spaces in a systematic manner. It is this systematic nature of the formalism that significantly simplifies the analysis or synthesis attempts in both circuit categories. Identifying and setting the BC as the circuit's central point, its analysis unfolds conveniently, regardless of the order or complexity of the system's equations. The scope of this tutorial paper is to expand and enrich the BCF and apply the outcome of this endeavor on the promising synaptic computation circuit field. In neural networks, synapses are important, key elements regarding information, computation and transmission.

Given the importance of these specialized biological structures, major effort has been put regarding the implementation of single synapses or synaptic networks by means of aVLSI circuits. In this paper we revisit a number of proposed in the literature synaptic circuits and classify them according to the type of their innate Bernoulli Cell operator. With the help of this work it is genuinely hoped that the interested reader will develop a deep understanding for the functionality of this class of low-power circuits and will appreciate the systematic nature of the formalism by consolidating the advantages of using one single framework to describe multiple, different, but in principle similar, log-domain synaptic topologies. This alternative treatment of aVLSI synaptic circuit succeeds in unifying the past analysis approaches of the same circuits under a common aegis and underlines the tutorial value of this paper. Finally, in order to reason for the versatility of the GBCF and to highlight its comparative speed advantage as an analysis tool, an indicative, high-order log-domain circuit topology is drawn from the international literature and is analyzed using both the GBCF and another common log-domain circuit analysis method. The compelling comparison results stress the advantages of using a single mathematical formalism for the description of any log-domain circuit, regardless of its linearity or order of complexity.

## 2. Expanding the bernoulli cell formalism

We start our mathematical analysis by mentioning briefly the equations that are characterizing an emitter/source connected linear capacitor and a BJT/MOST. Thereafter, the base/gate connected linear capacitor case is shown. It is important to stress at this point that the following analysis has been made for an npn-BJT and an n-type MOST. It has been left to the interested reader to verify the existence of a BC-operator in the case, when a pnp-BJT as well as a p-type MOST are emitter/source-connected to a linear capacitor. Further information can be found in the analysis here (Papadimitriou and Drakakis, 2012; Papadimitriou et al., 2013).

Furthermore, in all cases below, it has been assumed that the other plate of the capacitor is held at constant zero voltage (ground). Again, the reader can verify the existence of a BC-operator, when the capacitor's other plate is held at a random constant voltage, *V_DD_*, in all types of transistors. For the MOST analysis, we set the substrate-source voltage (*V_BS_*) equal to zero to achieve approximately the ideal exponential behavior by eliminating the “*body effect*.” Finally, all MOSTs are assumed to be in deep saturation, so that all transistors are operating qualitatively as constant current sources.

### 2.1. Emitter/source-connected capacitor BC topology

In the past work of Drakakis (Drakakis et al., 1997b, 1999a,b; Drakakis and Payne, 2000b), an explicit analysis has been illustrated regarding the current relation between an emitter-connected capacitor and a BJT. A similar analysis has been also presented regarding the current relation between a weakly-inverted MOST and a source-connected capacitor in Papadimitriou and Drakakis (2012); Papadimitriou et al. (2013). Both analyses led to the existence of a similar BC-operator and consequently are defined by a similar set of equations. The Bernoulli differential equations of the collector and drain currents of the aforementioned cases are shown below:

(1) $$\begin{array}{l} BJT\;Case:\\ {\dot{I}}_{C}(t)-\left(\frac{{\dot{V}}_{B}(t)}{U_{T}}+\frac{\left[u(t)-v(t)\right]}{CU_{T}}\right)I_{C}(t)+\frac{I_{C}^{2}(t)}{CU_{T}}=0 \end{array}$$

(2) $$\begin{array}{l} Subthreshold\;MOST\;Case:\\ {\dot{I}}_{D}(t)-\left(\frac{{\dot{V}}_{G}(t)}{nU_{T}}+\frac{\left[u(t)-v(t)\right]}{nCU_{T}}\right)I_{D}(t)+\frac{I_{D}^{2}(t)}{nCU_{T}}=0, \end{array}$$

where *U_T_* denotes the thermal voltage (~26 mV at 300 K) and *n* is the MOST's slope factor (*n* = 1/κ). In both cases, the currents *u*(*t*) and *v*(*t*) are the input and output currents of the aforementioned BC-operator (Drakakis et al., 1997b, 1999a,b; Drakakis and Payne, 2000b; Papadimitriou and Drakakis, 2012; Papadimitriou et al., 2013). By applying the non-linear substitution *I_C_*(*t*)=1/*T*(*t*)=*I_D_*(*t*), relations (1) and (2) are transformed into the following linearised form:

(3) $$\begin{array}{l} BJT\;Case:\\ \dot{T}(t)+\left(\frac{{\dot{V}}_{B}(t)}{U_{T}}+\frac{\left[u(t)-v(t)\right]}{CU_{T}}\right)T(t)-\frac{1}{CU_{T}}=0 \end{array}$$

(4) $$\begin{array}{l} Subthreshold\;MOST\;Case:\\ \dot{T}(t)+\left(\frac{{\dot{V}}_{G}(t)}{nU_{T}}+\frac{\left[u(t)-v(t)\right]}{nCU_{T}}\right)T(t)-\frac{1}{nCU_{T}}=0. \end{array}$$

The essence of the usefulness of the linearised forms of the BCs is located in the versatility that they provide, when the transfer function of a system is required (Drakakis et al., 1997b, 1999a,b; Drakakis and Payne, 2000b).

### 2.2. Base/gate-connected capacitor BC topology

**Figure 1:**
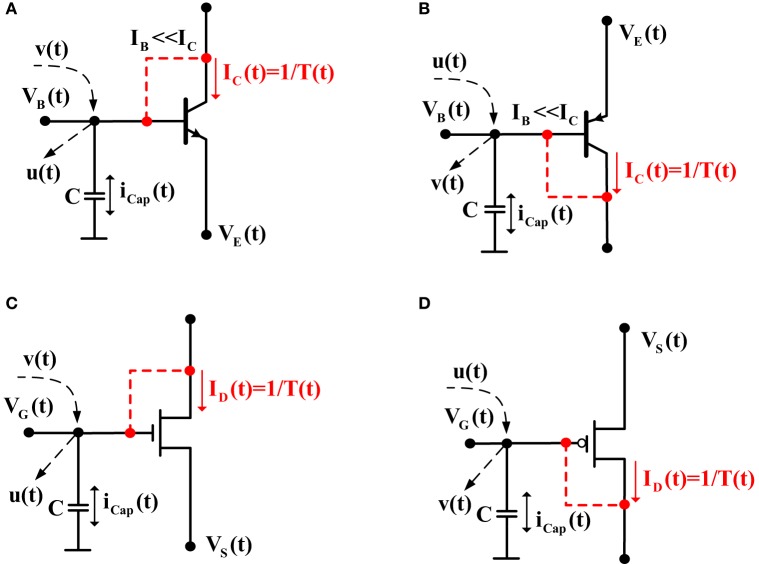
**Base/gate-connected capacitors to npn-, pnp-BJTs and n-, p-MOSTs that consist the new BC operator**. The arrows defining the direction of the capacitor current are bidirectional, since the BC analysis holds, whether the capacitor is connected to ground or *V_DD_*. The dashed lines reveal the diode-connected transistor case. Although the base current has been assumed to be significantly smaller than the collector current, the interested reader can verify even if it is comparable to the collector current value, it can still be assumed as a part of the *u*(*t*) output current. **(A)** An npn-BJT-based BC operator; **(B)** A pnp-BJT-based BC operator; **(C)** An n-MOST-based BC operator; **(D)** A p-MOST-based BC operator.

A closer look at the transistor-capacitor connections in Figure 1 will illustrate the existence of a different—in principle—BC operator. In this case, there are two possible circuital/connection combinations between the base/gate connected capacitor and the BJT/MOST. The first case, which is going to be explicitly analyzed in the following paragraphs, is the “*diode-connected*” topology. The second case, which is a subcategory of the first one (and not a focal point of this paper), is when the transistor's current is not responsible for the capacitor's charging and discharging behavior. Both cases will be mathematically analyzed, however, the first case is the most common one that directly exploits the Bernoulli differential equation to describe the transistor's current.

A diode-connected capacitor topology for a BJT is presented in Figures 1A,B, while for a subthreshold MOST the topology is shown in Figures 1C,D. For the BJT case analysis, we have assumed that the base current of the device is negligible compared to its collector current by considering very large values of β. However, it can be easily verified that even when the base current value is comparable to the collector current, it can be interpreted as one of the input/output currents of the BC topology and be assimilated into them. The following analysis will take place for a diode-connected npn-BJT and an n-type MOST.

Applying KCL at the capacitor node (see Figures 1A,C), we obtain for the input/output currents of the BC: *v*(*t*) = *u*(*t*)+ *I_C,D_*(*t*) + *i_Cap_*(*t*), for the BJT and MOST case, respectively. In both cases, it holds that the capacitor current *i_Cap_*(*t*) is equal to *C*$\dot{V}$_*B,G*_. By differentiating the ideal expressions of *I_C_* and *I_D_*, as explicitly shown in Drakakis et al. (1997b, 1999a), Drakakis et al. (1999b); Drakakis and Payne (2000b) the following Bernoulli differential equations are generated:

(5) $$\begin{array}{l} BJT\;Case:\\ {\dot{I}}_{C}(t)+\left(\frac{{\dot{V}}_{E}(t)}{U_{T}}+\frac{\left[u(t)-v(t)\right]}{CU_{T}}\right)I_{C}(t)+\frac{I_{C}^{2}(t)}{CU_{T}}=0 \end{array}$$

(6) $$\begin{array}{l} Subthreshold\;MOST\;Case:\\ {\dot{I}}_{D}(t)+\left(\frac{{\dot{V}}_{S}(t)}{nU_{T}}+\frac{\left[u(t)-v(t)\right]}{nCU_{T}}\right)I_{D}(t)+\frac{I_{D}^{2}(t)}{nCU_{T}}=0. \end{array}$$

By applying the non-linear substitution *I_C_*(*t*) = 1/*T*(*t*) = *I_D_*(*t*), as shown before, (5) and (6) are converted into the following linearised form:

(7) $$\begin{array}{l} BJT\;Case:\\ \dot{T}(t)-\left(\frac{{\dot{V}}_{E}(t)}{U_{T}}+\frac{\left[u(t)-v(t)\right]}{CU_{T}}\right)T(t)-\frac{1}{CU_{T}}=0 \end{array}$$

(8) $$\begin{array}{l} Subthreshold\;MOST\;Case:\\ \dot{T}(t)-\left(\frac{{\dot{V}}_{S}(t)}{nU_{T}}+\frac{\left[u(t)-v(t)\right]}{nCU_{T}}\right)T(t)-\frac{1}{nCU_{T}}=0. \end{array}$$

The interested reader should note that relations (5–8) exhibit striking similarities compared to the relations (1–4) that hold for the original BC operator case. One of the differences between the relations that describe the two BC operators is located at the sign after the time derivative of the state variable current, i.e. *İ_D_* or *İ_C_*. Moreover, relations (5–8) demonstrate a dependence on the time derivative of the source/emitter terminal's voltage of the MOST/BJT rather than on the time derivative of the gate/base terminal's voltage, as (1–4) do. It needs to be mentioned that the analysis holds, whether the capacitor is connected to ground or *V_DD_*. Finally, regarding the PMOS- or pnp-based “*diode-connected*” BC operator, trivial circuit analysis will reveal that the sign of the parameter $\dot{V}$_*S*_ or $\dot{V}$_*E*_ will change. Once again, all terms of equation (5) and (6) (and consequently 7–8) can be calculated by examining the currents that enter and/or leave at the capacitor node of the BC, except of the term $\dot{V}$_*S*_(*t*) or $\dot{V}$_*E*_(*t*), which primarily depends on the overall circuit's setup, as it will be revealed later.

The existence of the new BC operator mainly depends on the existence of a “*diode connection*” in the MOST or BJT device. When a “*diode connection*” is present, the state variable current *I_D_*(*t*) of the MOST (or *I_C_*(*t*) of the BJT) will be responsible for the charging or discharging phases of the capacitor. If one assumes that in the topologies shown in Figure 1, the “*diode connections*,” denoted by the red, dashed lines, are absent, then the transistor's current will not be involved in the KCL at the capacitor node and relations (5) and (6) are transformed into the following equations:

(9) $$\begin{array}{l} BJT\;Case:\\ {\dot{I}}_{C}(t)+\left(\frac{{\dot{V}}_{E}(t)}{U_{T}}+\frac{\left[u(t)-v(t)\right]}{CU_{T}}\right)I_{C}(t)=0 \end{array}$$

(10) $$\begin{array}{l} Subthreshold\;MOST\;Case:\\ {\dot{I}}_{D}(t)+\left(\frac{{\dot{V}}_{S}(t)}{nU_{T}}+\frac{\left[u(t)-v(t)\right]}{nCU_{T}}\right)I_{D}(t)=0. \end{array}$$

Relations (9) and (10) are not of the Bernoulli form, however, this type of connection can consist a subcategory of the new “*diode connection*” BC operator case. If one of the input/output currents of the BC is a function of the state-variable current, i.e. if *u_j_* (*and/or v_j_*) = 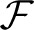
(*I_C,D_*), then the Bernoulli differential equation is constructed again. A typical circuit case that verifies this subcategory of the BC topology is the log-domain synaptic circuit originally proposed in Shi and Horiuchi (2004).

## 3. Exemplary synaptic circuits' analyses based on the generalized BC formalism

An interesting application, on which the GBCF could be applied, is the popular subcategory of neuromorphic circuits, the silicon synaptic circuits. In neural networks, synapses consist important, key elements regarding information computation and transmission (Bartolozzi and Indiveri, 2007). Given the importance of these specialized structures, major effort has been made regarding the implementation of single synapses or synaptic networks by means of aVLSI circuits. By exploiting the exponential current-voltage relation of weakly-inverted MOSTs a wide variety of circuits has been implemented, capable of simulating different types of synaptic behaviors.

Silicon synapses are able to transform a voltage pulse, which simulates a pre-synaptic signal, into post-synaptic currents that stimulate the membrane of targeted neighboring neurons. Moreover, the gain of such post-synaptic signal, usually referred as synaptic weight, can be also introduced by the specific circuits by simply altering specific electrical parameters, which correspond to equivalent biological parameters (Liu et al., 2001; Bartolozzi and Indiveri, 2007). As these circuits are usually very compact in size, the implementation of very large synaptic networks is possible.

In the following paragraphs, an indicative number of synaptic circuits is going to be analyzed based on the proposed formalism, proving the systematic nature of the GBCF. The selection of the presented circuits is only based on their popularity and extensive use by the neuromorphic community, as well as on their relatively complicated nature, compared to other similar circuits in this category (Bartolozzi and Indiveri, 2007; Indiveri et al., 2011).

### 3.1. Log-domain integrator synapse

In the tutorial paper of Bartolozzi and Indiveri (Bartolozzi and Indiveri, 2007) a useful synaptic circuit is presented, called “*Log-Domain Integrator Synapse*” (LDI). The properties of this linear integrator circuit are explicitly presented in Bartolozzi and Indiveri (2007) as well as in the original publications (Merolla and Boahen, 2004, 2006; Arthur and Boahen, 2006) and are similar to the linear properties of a log-domain filter. The *M_pre_* transistor is triggered by a sequence of voltage pulses, where *t*^−^ is the time at which the *i^th^* input spike arrives and *t*^+^ is the time at which it ends.

**Figure 2:**
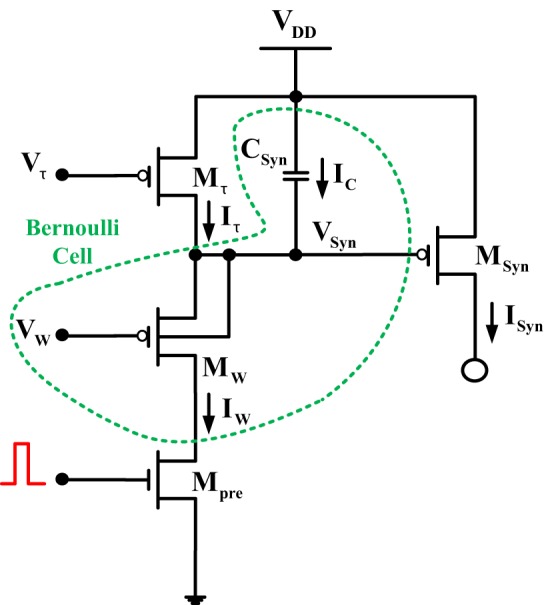
**Log-domain integrator synapse**. The dashed circular area is enclosing the BC-operator for this circuit.

In order to start the BC-based circuit analysis, it is important to identify first the BC-operator of the given topology. In this case, the BC-operator is enclosed by the dashed green line (see Figure 2). Applying KCL at the capacitor node *V_Syn_*(*t*) reveals: *I_W_*(*t*) = *I*_τ_(*t*) + *I_C_*(*t*) with *I_C_*(*t*) been equal to −*C*$\dot{V}$_*Syn*_(*t*). The relation between the state variable current of the BC, *I_W_*(*t*) and the output current of the circuit *I_Syn_*(*t*) can be easily determined:

$$\begin{array}{l} I_{W}(t)=I_{O}\;e^{\frac{V_{Syn}(t)-V_{W}(t)}{nU_{T}}}\\ I_{Syn}(t)=I_{O}\;e^{\frac{V_{DD}-V_{Syn}(t)}{nU_{T}}} \end{array}\}\Rightarrow I_{W}(t)I_{Syn}(t)=I_{O}I_{WO},$$

where the current *I_WO_* is defined as *I_O_ exp*(− (*V_W_*(*t*) − *V_DD_*)/(*nU_T_*) with *I_O_* denoting the leakage current of the transistors. The current *I_WO_* designates the initial current that flows through the transistor *M_W_* when *V_DD_* = *V_Syn_*(*t*) (Bartolozzi and Indiveri, 2007). By differentiating *I_W_*(*t*) with respect to time, it yields: 
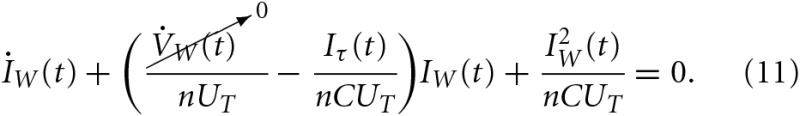


Since *V_W_* is a constant bias voltage, its time derivative should be equal to zero, therefore:

(12) $${\dot{I}}_{W}(t)-\frac{I_{\tau }(t)}{nCU_{T}}I_{W}(t)+\frac{I_{W}^{2}(t)}{nCU_{T}}=0.$$

The time behavior of the state variable current of the BC *I_W_*(*t*) is governed by the Bernoulli differential equation. Moreover, based on the relation between the currents *I_W_*(*t*) and *I_Syn_*(*t*), and between *I_W_*(*t*) and *I*_τ_(*t*), (12) can be re-written as:

(13) $$\tau {\dot{I}}_{Syn}(t)+I_{Syn}(t)=\frac{I_{O}I_{WO}}{I_{\tau }(t)},$$

with τ = *nCU_T_*/*I*_τ_(*t*). The explicit solution of (13) must be separated into two different phases: (a) charge phase of the capacitor, where input current enters the BC and (b) discharge phase of the capacitor, where no input current enters the BC. Summing up both solutions for both phases, the following expressions for the output current *I_Syn_*(*t*) are obtained:

$$I_{Syn}(t)=\{\begin{array}{ll} & Charge\;phase:\\ & \frac{I_{O}I_{WO}}{I_{\tau }(t)}\left(1-e^{\frac{-\left(t-t_{i}^{-}\right)}{\tau }}\right)+I_{Syn}^{-}e^{\frac{-\left(t-t_{i}^{-}\right)}{\tau }}\\ & Discharge\;phase:\\ & I_{Syn}{\left(t\right)}^{+}e^{-\frac{\left(t-t_{i}^{+}\right)}{\tau }}. \end{array}$$

### 3.2. Differential pair integrator synapse

The “Differential-Pair Integrator (DPI) Synapse” was firstly presented in the same tutorial paper of Bartolozzi and Indiveri (Bartolozzi and Indiveri, 2007) in 2007 and is able to reproduce the exponential dynamics observed in both excitatory and inhibitory post-synaptic currents of biological synapses. The idea behind the design of such a circuit is the development of a topology, which maintains its filtering properties while overcoming the LDI's shortcoming of generating sufficiently large charge packets sourced into the capacitor for brief input spikes. The DPI synapse does not require any additional pulse-extender circuits and in addition it can be manufactured without requiring isolated well structures.

**Figure 3:**
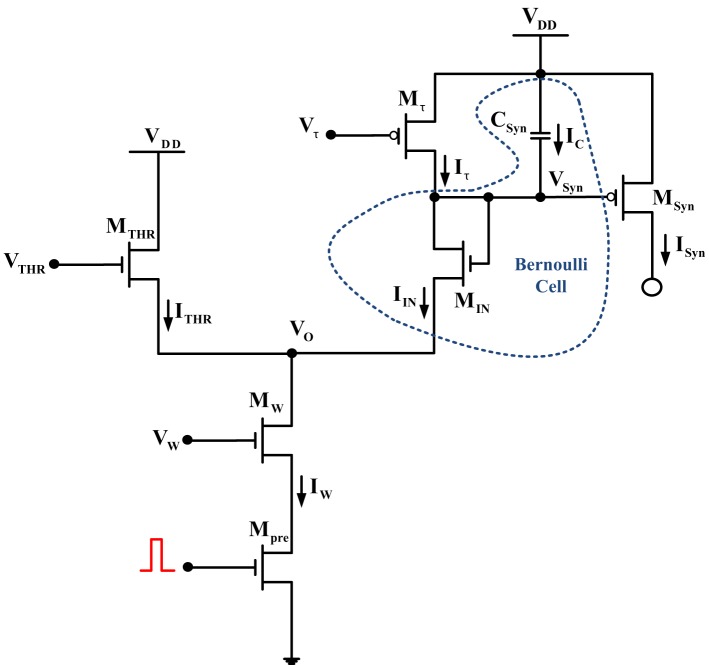
**Differential pair integrator synapse**. The dashed circular area is again enclosing the BC-operator for this circuit.

A detailed analysis of this circuit is sufficiently presented in Bartolozzi and Indiveri (2007). Following a series of well-based hypotheses, the authors conclude to the following differential equation expression of the output current of the circuit, *I_Syn_*(*t*) (see Figure 3):

(14) $$\tau {\dot{I}}_{Syn}(t)+I_{Syn}(t)=\frac{I_{W}(t)I_{Gain}(t)}{I_{\tau }(t)},$$

where the term *I_Gain_*(*t*) = *I_O_ exp*(− (*V_DD_* − *V_THR_*)/(*nU_T_*)) represents a virtual p-type MOST and τ = *nCU_T_*/*I*_τ_(*t*). The logic assumptions leading to (14) are: *I_W_* ≫ *I*_τ_ and *I_Syn_* ≫ *I_Gain_*. Based on these assumptions, it is obvious that (14) implements a first order equation similar to one presented for the LDI circuit. The interested reader should note the resemblance between the solutions of the DPI and LDI synaptic circuits. The only fundamental difference, from a mathematical point of view, is that the current *I_O_* has been replaced by the current of the virtual MOST *I_Gain_*.

For the BC-analysis of the DPI circuit, a similar systematic analysis approach will be applied as in the previous example. The BC has been identified and encircled by the blue dashed line (see Figure 3). Applying KCL at node *V_Syn_*(*t*) shows that:

$$I_{IN}(t)=I_{\tau }(t)+I_{C}(t)\Leftrightarrow {\dot{V}}_{Syn}(t)=(I_{\tau }(t)-I_{IN}(t))/C,$$

where *I_C_*(*t*) = −*C*$\dot{V}$_*Syn*_(*t*). Considering the drain current of the diode-connected BC transistor:

$$I_{IN}(t)=I_{O}\;exp((V_{Syn}(t)-V_{O}(t)/(nU_{T}))$$

as the circuit's state variable and by differentiating it with respect to time, it yields:

(15) $${\dot{I}}_{IN}(t)+\left(\frac{{\dot{V}}_{O}(t)}{nU_{T}}-\frac{I_{\tau }(t)}{nCU_{T}}\right)I_{IN}(t)+\frac{I_{IN}^{2}(t)}{nCU_{T}}=0.$$

In order to create an ODE, where all factors can be computed, the time behavior of the term $\dot{V}$_*O*_(*t*) in (15) must be investigated. Starting from the well known relation that holds for the differential pair topology:

(16) $$I_{W}(t)e^{\frac{V_{O}(t)}{nU_{T}}}=I_{O}\left(e^{\frac{V_{Syn}(t)}{nU_{T}}}+e^{\frac{V_{THR}}{nU_{T}}}\right)$$

and by differentiating both sides of (16), it holds that: 
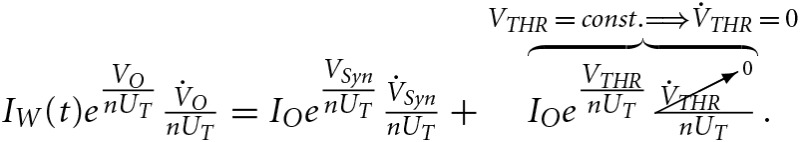


After the above treatment, it yields that $\dot{V}$_*O*_(*t*) = $\dot{V}$_*Syn*_(*t*)*I_IN_*(*t*)/*I_W_*(*t*). By substituting this expression into (15), we end up with the following form of ODE:

$${\dot{I}}_{IN}(t)+\frac{I_{IN}^{2}(t)I_{\tau }(t)}{I_{W}(t)nCU_{T}}-\frac{I_{IN}^{2}(t)\overset{I_{W}(t)-I_{THR}(t)}{\overset{︷}{I_{IN}(t)}}}{I_{W}(t)nCU_{T}}+\frac{I_{IN}^{2}(t)}{nCU_{T}}-\frac{I_{IN}(t)I_{\tau }(t)}{nCU_{T}}=0$$

or equivalently:

(17) $${\dot{I}}_{IN}(t)-\frac{I_{IN}(t)I_{\tau }(t)}{nCU_{T}}+\frac{I_{IN}^{2}(t)}{nCU_{T}}\left[\frac{I_{\tau }(t)}{I_{W}(t)}+\frac{I_{THR}(t)}{I_{W}(t)}\right]=0.$$

Based on the valid assumptions that the authors did in Bartolozzi and Indiveri (2007), it holds that *I_W_* ≫ *I*_τ_ and also *I_THR_* ≈ *I_W_*, thus, (17) is finally transformed into:

(18) $${\dot{I}}_{IN}(t)-\frac{I_{IN}(t)I_{\tau }(t)}{nCU_{T}}+\frac{I_{IN}^{2}(t)}{nCU_{T}}=0.$$

Equation 18 is a Bernoulli ODE with respect to *I_IN_*(*t*) and can be solved by using the usual non-linear transformation. A brief mathematical explanation why *I_THR_* ≈ *I_W_* is provided in the Appendix of the paper. In the final solution of *I_IN_*(*t*), we can select to substitute *I_IN_*(*t*) with its equivalent equation which includes *I_Syn_*(*t*). This equivalent expression is derived as follows from the differential pair's key equation:

(19) $$I_{IN}(t)=\frac{I_{W}(t)exp\left(V_{Syn}(t)/(nU_{T})\right)}{exp(V_{Syn}(t)/(nU_{T}))+exp\left(V_{THR}/(nU_{T})\right)}$$

and by multiplying both the numerator and denominator by *exp*(− *V_DD_*/(*nU_T_*)), it is easy to express *I_IN_* as:

(20) $$I_{IN}(t)=\left(I_{W}(t)I_{Gain}(t)\right)/(I_{Gain}(t)+I_{Syn}(t)),$$

where *I_Gain_* has been defined above. Therefore, if (20) is placed into the explicit solution of (18) and bearing in mind that *I_Syn_* ≫ *I_Gain_*, the final expressions for the current *I_Syn_*(*t*) during charge and discharge phases are described below:

$$I_{Syn}(t)=\{\begin{array}{l} Charge\;phase:\\ \frac{I_{Gain}(t)I_{W}(t)}{I_{\tau }(t)}\left(1-e^{-\frac{\left(t-t_{i}^{-}\right)}{\tau }}\right)+I_{Syn}^{-}e^{-\frac{\left(t-t_{i}^{-}\right)}{\tau }}\\ Discharge\;phase:\\ I_{Syn}^{+}e^{-\frac{\left(t-t_{i}^{+}\right)}{\tau }}. \end{array}$$

It has been left to the reader again to verify that the above solution is similar to the one presented in the original paper, derived for a sequence of voltage pulses with τ = *nCU_T_*/*I*_τ_(*t*).

## 4. Current-mode circuits for depressing and facilitating synapses implementation

Dynamical synapses can be depressing, facilitating or even a combination of theses two (Liu, 2003). aVLSI circuits implementing depressing and facilitating synaptic behaviors have been extensively presented and analyzed in literature (Rasche and Hahnloser, 2001; Liu, 2003). In this paper, due to lack of space reasons, only the mathematical description of a facilitating synaptic circuit will be presented. An identical analysis holds for the description of a circuit emulating a depressing synaptic behavior (Liu, 2003).

### 4.1 BC-based analysis of a facilitating synapse circuit

**Figure 4:**
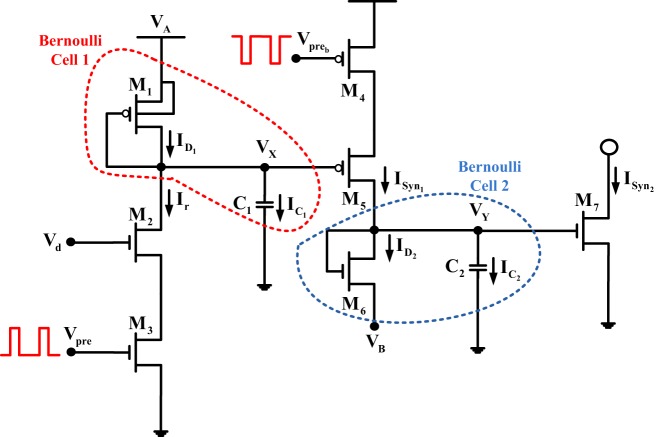
**Synaptic facilitation circuit**. The red and blue dashed lines define the BC-operators of the circuit.

A typical configuration of a circuit implementing a facilitating silicon synapse is the one shown in Figure 4. From this Figure one can identify two, distinct “*circuit stages*,” due to the existence of the two capacitors. In each one of these stages a BC-operator can be identified and will be analyzed separately below. The first BC operator is encircled by the red dashed line, while the second operator is encircled by the blue dashed line.

• **BC-1:** KCL at node *V_X_* yields that *I*_*D*_1__(*t*) = *I_r_*(*t*) + *I*_*C*_1__(*t*), where the capacitor current *I*_*C*_1__(*t*) can be also defined as *C*_1_$\dot{V}$_*X*_ or $\dot{V}$_*X*_(*t*) = (*I*_*D*_1__(*t*) −*I_r_*(*t*))/*C*_1_. The time-derivative of the state variable current of the first BC *I*_*D*_1__(*t*) = *I_O_ exp*((*V_A_* − *V_X_*)/(*nU_T_*)) will be:

(21) $${\dot{I}}_{D_{1}}(t)-\frac{I_{r}(t)}{nC_{1}U_{T}}I_{D_{1}}(t)+\frac{I_{D_{1}}^{2}(t)}{nC_{1}U_{T}}=0.$$

As expected, (21) is the Bernoulli ODE governing the drain current dynamics of the diode-connected MOST *M*_1_. For the calculation of the output current of the first “*stage*” of the circuit, we need to find the relation between *I*_*D*_1__(*t*) and *I*_*Syn*_1__(*t*) and substitute it back to (21). The relation between these two currents can be easily derived by writing their full exponential expressions: 
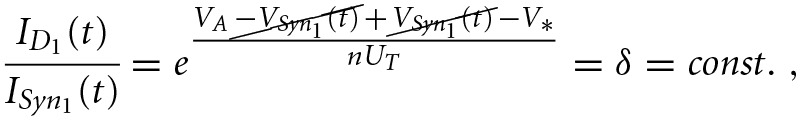
 where V_*_ is the source voltage of *M*_5_, which as illustrated in Figure 4 is constant. Therefore, (21) transforms into:

(22) $${\dot{I}}_{Syn_{1}}(t)-\frac{I_{r}(t)}{nC_{1}U_{T}}I_{Syn_{1}}(t)+\frac{\delta I_{Syn_{1}}^{2}(t)}{nC_{1}U_{T}}=0.$$

• **BC-2:** For the *BC*_2_, KCL at the capacitor node *V_Y_* shows that: *I*_*D*_2__(*t*) + *I*_*C*_2__(*t*) = *I*_*Syn*_1__(*t*). Moreover, the capacitor current *I*_*C*_2__(*t*) can be also defined as *C*_2_$\dot{V}$_*Y*_, which finally gives $\dot{V}$_*Y*_(*t*) = (*I*_*Syn*_1__(*t*) − *I*_*D*_2__(*t*))/*C*_2_. The derivative of the state variable current of the *BC*_2_, *I*_*D*_2__(*t*) = *I_O_ exp*((*V_Y_*(*t*) − *V_b_*)/(*nU_T_*)) yields:

(23) $${\dot{I}}_{D_{2}}(t)-\frac{I_{Syn_{1}}(t)}{nC_{2}U_{T}}I_{D_{2}}(t)+\frac{I_{D_{2}}^{2}(t)}{nC_{2}U_{T}}=0.$$

Again, relation (23) identifies the Bernoulli ODE dynamics of the diode-connected MOST *M*_6_. Moreover, in this “*stage*” of the circuit, the relation between *I*_*D*_2__(*t*) and *I*_*Syn*_2__(*t*) is given by the following equation: 
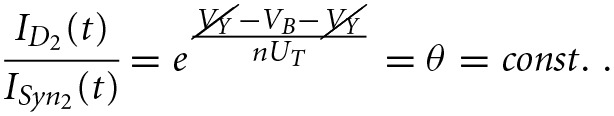


Thus, the new ODE for the output synaptic current *I*_*Syn*_2__(*t*) can be calculated by:

(24) $${\dot{I}}_{Syn_{2}}(t)-\frac{I_{Syn_{1}}(t)}{nC_{2}U_{T}}I_{Syn_{2}}(t)+\frac{\theta I_{Syn_{2}}^{2}(t)}{nC_{2}U_{T}}=0.$$

At this point it would be useful to stress that relation (24) is a BC-cascaded relation, where the output of the first BC is included in the differential equations of the second BC, as a *v*(*t*) current.

## 5. Comparative analysis of a log-domain topology with a high number of bernoulli cells - the “speed up” impact of the formalism

The previous Sections (3 and 4) have proven the reasons why the core dynamics of three well-known log-domain synaptic circuits proposed by different researchers comply with the same distinct Bernoulli dynamics in a formal manner. This mathematical fact alone offers deep and unifying insight since many neuromorphic circuits can be described by the GBCF (see later **Table 2**). It can be argued that the Bernoulli dynamics constitute a formal insightful re-expression of KCL when the derivation of the specific differential equation (which considers the application of KCL at the capacitor node) is born in mind. Such a re-expression is directly applicable/exploitable in a purely TL environment; the same TL environment for which the celebrated Gilbert's TLP can be viewed as a profound re-expression of KVL which has led to the conception of many new, mostly non-linear, monolithic circuits.

**Figure 5:**
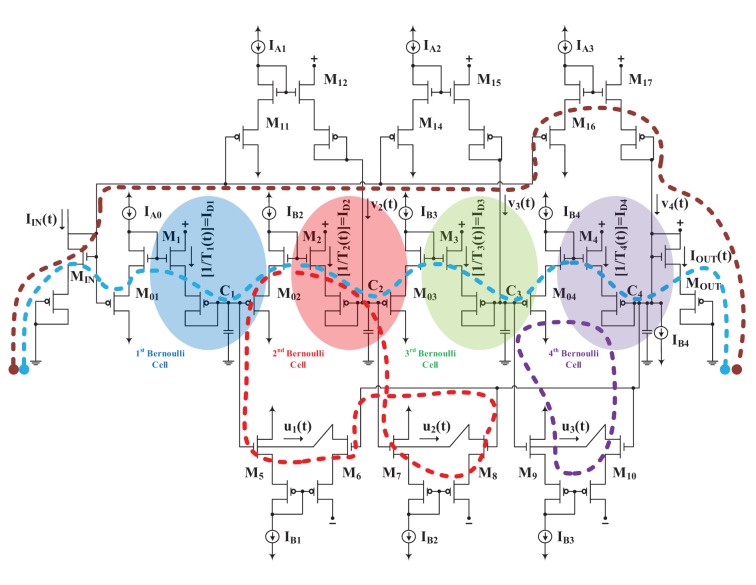
**CMOS version of the BJT log-domain topology proposed by Wu and El-Masry (1998)**. The topology contains four compound (each composed of two *V_GS_*) Bernoulli Cells and a multitude of complete TL loops (some are indicatively depicted by means of dashed lines).

Apart from being useful as a taxonomy tool and apart from facilitating researchers to comprehend the essence of the functionality of various log-domain synaptic circuits, is there any additional practical advantage when adopting the Bernoulli Cell formalism? Experience reveals that the higher the number of Bernoulli Cells present in a log-domain topology be it a nonlinear (such as a synapse) or an ELIN one, the faster and less prone to errors its hand-analysis becomes. In order to exemplify vividly how sped up the analysis becomes, in this section we analyse a high-order ELIN log-domain topology both by the general method proposed by Mulder (Mulder et al., 1997; Mulder, 1998) and by the GBCF. The example topology, shown in Figure 5, contains four BCs and has been proposed in the international literature by Wu and El-Masry in Wu and El-Masry (1998). In its original form it involved only BJT devices. Here we have substituted the BJT devices for MOSTs and we assume that the n- and p- devices are identical in size and physical properties. This maintains the complexity of the analyses manageable and, most importantly, thus serves the tutorial character of this paper. The aim of this section of the paper is to provide compelling comparative analysis results which shed light in a tutorial manner on “*how much*” the GBCF speeds up the analysis of a log-domain topology which contains many BCs.

Referring to Figure 5 and before proceeding with its analysis, it should also be noted that we assume that the dc biasing currents of the circuit are of such values that each device in the circuit will have a valid dc operating point. This translates into the satisfaction of certain biasing constraints (Drakakis and Burdett, 2003). According to (Mulder et al., 1997; Mulder, 1998), the following steps are necessary, in order to analyse any log-domain topology and derive the relationship between the input and the output (input-output transfer function) in the case of “*Externally-Linear-Internally-Nonlinear*” log-domain topologies^1^:

**Step 1:** The application of KCL at the integrating nodes of the log-domain topology in question must be considered; the capacitor currents are treated as unknowns.
**Step 2:** The application of the TLP along convenient TL loops must be considered.
**Step 3:** The capacitor currents (the unknowns) must be derived in terms of other currents in the circuit; this can be done by means of mesh analysis treating a capacitor voltage *V_C_j__*(*t*) and a certain number of MOSTs (gate-source voltage difference) in series with the capacitor as a loop. Expressing *V_C_j__*(*t*) in terms of the MOST terminal voltages within this loop leads to an equation of the form:
  $$V_{C_{j}}(t)=nU_{T}\sum \pm ln\left[\frac{I_{D_{j}}(t)}{{\left[W/L\right]}_{j}I_{D_{O}}}\right],$$
  with the drain currents, the process parameter *I_D_O__* and the aspect ratios of the transistors involved, respectively. Once this has taken place the capacitor currents can be expressed as:
  $$i_{C_{j}}(t)=C_{j}{\dot{V}}_{C_{j}}(t)=nC_{j}U_{T}\sum_{j}\pm \frac{{\dot{I}}_{D_{j}}(t)}{I_{D_{j}}(t)}.$$
  Relations of this kind are then used to eliminate the capacitor currents derived during steps 1 and 2; *to derive the final transfer function all capacitor currents must be expressed in terms of the input the output and their derivatives*.

Applying step 1, it can be observed that:

(25a) $$I_{D_{1}}(t)=u_{1}(t)+i_{C_{1}}(t)$$

(25b) $$I_{D_{2}}(t)=u_{2}(t)-v_{2}(t)+i_{C_{2}}(t)$$

(25c) $$I_{D_{3}}(t)=u_{3}(t)-v_{3}(t)+i_{C_{3}}(t)$$

(25d) $$I_{D_{4}}(t)=u_{4}(t)-v_{4}(t)+i_{C_{4}}(t),$$

where *I_D_j__*(*t*), (*j* = 1,..4) the drain current of *M_j_*, (*j* = 1,..4). For step 2, applying the TLP along the complete TL loops: *M*_02_*M*_2_*M*_7_*M*_8_*M*_6_*M*_5_, *M*_7_*M*_8_*M*_10_*M*_9_*M*_3_*M*_03_, *M*_9_*M*_10_*M*_4_*M*_04_, *M_IN_M*_01_*M*_1_*M*_02_*M*_2_*M*_03_*M*_3_*M*_04_*M*_4_*M_OUT_* will lead to the following Translinear current relationships (in Figure 5 we mark indicatively a few of the TL loops present to make the analysis more vivid):

(26a) $$u_{1}(t)I_{B2}\left[u_{2}(t)-v_{2}(t)+i_{C_{2}}(t)\right]=I_{B1}I_{B2}u_{2}(t)$$

(26b) $$u_{2}(t)I_{B3}\left[u_{3}(t)-v_{3}(t)+i_{C_{3}}(t)\right]=I_{B2}I_{B3}u_{3}(t)$$

(26c) $$u_{3}(t)\left[I_{B4}-v_{4}(t)+i_{C_{4}}(t)\right]=I_{B3}I_{B4}$$

(26d) $$\begin{array}{l} I_{IN}I_{A0}I_{B2}I_{B3}I_{B4}=\left[u_{1}(t)+i_{C_{1}}(t)\right]\left[u_{2}(t)-v_{2}(t)+i_{C_{2}}(t)\right]\\ \left[u_{3}(t)-v_{3}(t)+i_{C_{3}}(t)\right]\left[I_{B4}-v_{4}(t)+i_{C_{4}}(t)\right]I_{OUT}. \end{array}$$

Proceeding to step 3, the capacitor currents *i_C_j__*(*t*), (*j* = 1,..4) that have been treated as unknowns, now have to be related to other output currents in a convenient way. For the capacitor current *i*_*C*_4__(*t*), an elegant expression can be derived:

$$i_{C4}(t)=2nC_{4}U_{T}\frac{{\dot{I}}_{OUT}(t)}{I_{OUT}(t)}.$$

However, for the rest of the capacitor currents such a simple expression cannot be similarly obtained. Among the variety of possible ways in which to express the capacitor currents as functions of (internal) circuit currents, certain meshes which seem to result into simple expressions for the capacitor currents are chosen. Considering the paths *C*_3_*M*_9_*M*_10_*M*_*OUT*_, *C*_2_*M*_7_*M*_8_*M*_*OUT*_, and *C*_1_*M*_5_*M*_6_*M*_*OUT*_ leads to the following capacitor voltage and current relations, respectively:

(27a) $$\begin{array}{l} V_{C3}(t)=2nU_{T}ln\left[\frac{I_{B3}}{[W/L]I_{D_{O}}}\right]-2nU_{T}ln\left[\frac{u_{3}(t)}{[W/L]I_{D_{O}}}\right]\\ \;+2nU_{T}ln\left[\frac{I_{OUT}(t)}{[W/L]I_{D_{O}}}\right] \end{array}$$

(27b) $$\begin{array}{l} V_{C2}(t)=2nU_{T}ln\left[\frac{I_{B2}}{[W/L]I_{D_{O}}}\right]-2nU_{T}ln\left[\frac{u_{2}(t)}{[W/L]I_{D_{O}}}\right]\\ \;+2nU_{T}ln\left[\frac{I_{OUT}(t)}{[W/L]I_{D_{O}}}\right] \end{array}$$

(27c) $$\begin{array}{l} V_{C1}(t)=2nU_{T}ln\left[\frac{I_{B1}}{[W/L]I_{D_{O}}}\right]-2nU_{T}ln\left[\frac{u_{1}(t)}{[W/L]I_{D_{O}}}\right]\\ \;+2nU_{T}ln\left[\frac{I_{OUT}(t)}{[W/L]I_{D_{O}}}\right], \end{array}$$

which in turn leads to:

(28) $$i_{C\rho }=C_{\rho }{\dot{V}}_{C\rho }(t)=2nC_{\rho }U_{T}\left[\frac{{\dot{I}}_{OUT}(t)}{I_{OUT}(t)}-\frac{{\dot{u}}_{\rho }(t)}{u_{\rho }(t)}\right]\left(\rho =1,\;2,\;3\right)\text{​}.$$

As mentioned previously *i*_*C*4_(*t*) is in the right “*form*” being directly related to the output current. However, the other three capacitor currents are not in a desirable form; the currents *u*_3_(*t*), *u*_2_(*t*), *u*_1_(*t*) must first be expressed in terms of the input, the output and their derivatives. This procedure is cumbersome. In order to demonstrate the difficulty in expressing the aforementioned currents in the correct “*form*,” only the procedure to express the current *u*_3_(*t*) in terms of the input, the output and their derivatives will be demonstrated. Taking relation (26c) into consideration, *u*_3_(*t*) can be described as:

$$u_{3}(t)=\frac{I_{B3}I_{B4}}{I_{B4}-v_{4}(t)+i_{C4}(t)},$$

with *i*_*C*4_(*t*) given before. The current *v*_4_(*t*) however must also be written in a convenient format; considering the complete TL loop *M_IN_M*_16_*M*_17_*M_OUT_* yields:

$$I_{IN}(t)I_{A3}=v_{4}(t)I_{OUT}(t)\Rightarrow v_{4}(t)=I_{A3}\frac{I_{IN}(t)}{I_{OUT}(t)}.$$

From the previous relations for *i*_*C*4_(*t*) and *v*_4_(*t*), it is a matter of complicated algebraic substitutions to express the current *u*_3_(*t*) in the “*correct form*” (i.e. expressed in terms of the input current, the output current and their derivative) as: 
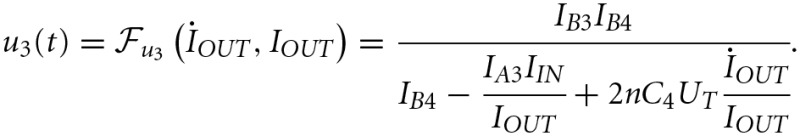


Substituting the relation for *u*_3_(*t*) into (28) (ρ = 3) results into the following expression, *just* for *i*_*C*3_(*t*):

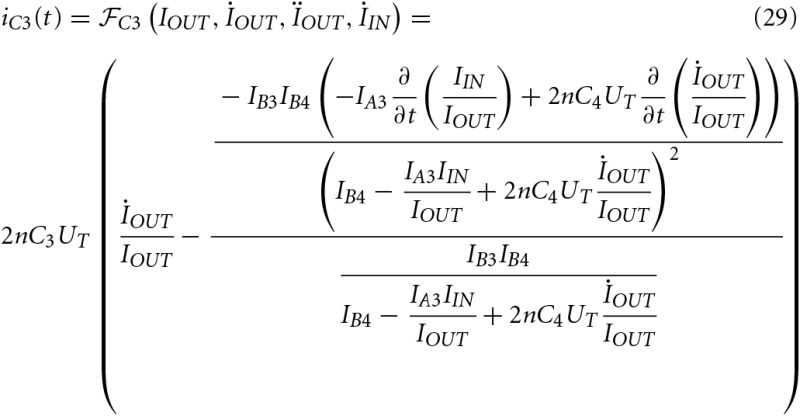



In a similar manner, all the remaining currents *i*_*C*2_(*t*), *i*_*C*1_(*t*), *u*_1_(*t*), *u*_2_(*t*) etc. need to be defined in the “*correct form*.” Having reached that point, we would finally need to substitute to, say, (26d) all the expressions of the “*correct form*” found and determine the input-output transfer function. Or one might choose to substitute all the “*correct form*” expressions to the TL equality:

$$I_{IN}(t)I_{A0}u_{1}(t)=\left[u_{1}(t)+i_{C1}(t)\right]I_{B1}I_{OUT}(t),$$

which correspond to the TL loop *M_IN_M*_01_*M*_1_*M*_5_*M*_6_*M_OUT_* and again determine the input-output transfer function.

Clearly this analysis procedure is tedious, time-consuming and prone to errors since all the “*intermediate*” *u_j_*(*t*) and *v_j_*(*t*) currents must be expressed in the right format. These requirements are compounded when large topologies with a large number of intermediate currents are considered, making the method difficult to apply as far as hand calculations are considered. At this point it is worth mentioning that the situation is somewhat improved, when a variation of the method is considered which consists of the incorporation of additional “*fictitious*” exponential expansion stages which convert the logarithmically compressed capacitor voltages *V*_*C*_1__(*t*), *V*_*C*_2__(*t*) and *V*_*C*_3__(*t*) to exponentially expanded currents, without affecting the circuit operation. These additional stages add complexity to the circuit but lead to the formation of three more, perhaps more convenient complete TL loops, which relate the “*fictitious*” output currents with the real output current *I_OUT_* shown in Figure 5. However, further elaboration on that analysis method is beyond the scope of this work.

Now let us analyse the same log-domain structure by means of the GBCF. Four distinct BCs can be identified; the first one is logarithmically driven by the input current *I_IN_*(*t*). This cascade of compound BCs can be described by means of the following LDSS equations, i.e.:

(30a) $$2nC_{1}U_{T}{\dot{w}}_{1}(t)+\left[u_{1}(t)-v_{1}(t)\right]w_{1}(t)=I_{IN}(t)$$

(30b) $$2nC_{2}U_{T}{\dot{w}}_{2}(t)+\left[u_{2}(t)-v_{2}(t)\right]w_{2}(t)=w_{1}(t)$$

(30c) $$2nC_{3}U_{T}{\dot{w}}_{3}(t)+\left[u_{3}(t)-v_{3}(t)\right]w_{3}(t)=w_{2}(t)$$

(30d) $$2nC_{4}U_{T}{\dot{w}}_{4}(t)+\left[u_{4}(t)-v_{4}(t)\right]w_{4}(t)=w_{3}(t).$$

The products *u_j_*(*t*)*w_j_*(*t*) and *v_j_*(*t*)*w_j_*(*t*) are determined as required by applying the TLP along complete TL loops. Considering the time-domain current product equalities resulting from the TL loops: (*i*) *M*_5_*M*_6_*M*_4_*M*_04_*M*_3_*M*_03_*M*_2_*M*_02_, (*ii*) *M*_7_*M*_8_*M*_4_*M*_04_*M*_3_*M*_03_, (*iii*) *M*_9_*M*_10_*M*_4_*M*_04_, (*iv*) *M*_01_*M*_1_*M*_02_*M*_2_*M*_12_*M*_11_, (*v*) *M*_01_*M*_1_*M*_02_*M*_2_*M*_03_*M*_3_*M*_15_*M*_14_, (*vi*) *M*_01_*M*_1_*M*_02_*M*_2_*M*_03_*M*_3_*M*_04_*M*_4_*M*_17_*M*_16_, and (*vii*) *M*_*IN*_*M*_01_*M*_1_*M*_02_*M*_2_*M*_03_*M*_3_*M*_04_*M*_4_*M_OUT_* yields respectively:

(31a) $$\begin{array}{l} u_{1}(t)w_{1}(t)=I_{B1}I_{B2}I_{B3}I_{B4}T_{4}(t)T_{3}(t)T_{2}(t)w_{1}(t)\\ \;=I_{B1}I_{B2}I_{B3}I_{B4}w_{4}(t) \end{array}$$

(31b) $$\begin{array}{l} u_{2}(t)w_{2}(t)=I_{B2}I_{B3}I_{B4}T_{4}(t)T_{3}(t)w_{2}(t)\\ \;=I_{B2}I_{B3}I_{B4}w_{4}(t) \end{array}$$

(31c) $$u_{3}(t)w_{3}(t)=I_{B3}I_{B4}T_{4}(t)w_{3}(t)=I_{B3}I_{B4}w_{4}(t)$$

(31d) $$v_{2}(t)T_{2}(t)T_{1}(t)I_{IN}(t)=v_{2}(t)w_{2}(t)=\frac{I_{A1}}{I_{A0}I_{B2}}I_{IN}(t)$$

(31e) $$\begin{array}{l} v_{3}(t)T_{3}(t)T_{2}(t)T_{1}(t)I_{IN}(t)=v_{3}(t)w_{3}(t)\\ \;=\frac{I_{A2}}{I_{A0}I_{B2}I_{B3}}I_{IN}(t) \end{array}$$

(31f) $$\begin{array}{l} v_{4}(t)T_{4}(t)T_{3}(t)T_{2}(t)T_{1}(t)I_{IN}(t)=v_{4}(t)w_{4}(t)\\ \;=\frac{I_{A3}}{I_{A0}I_{B2}I_{B3}I_{B4}}I_{IN}(t) \end{array}$$

(31g) $$\begin{array}{l} I_{OUT}(t)=I_{A0}I_{B2}I_{B3}I_{B4}T_{4}(t)T_{3}(t)T_{2}(t)T_{1}(t)I_{IN}(t)\\ \;=I_{A0}I_{B2}I_{B3}I_{B4}w_{4}(t). \end{array}$$

Substituting the relations (31a)–(31g) into the LDSS equations (30) results in the following system of differential equations:

(32a) $$2nC_{1}U_{T}{\dot{w}}_{1}(t)+I_{B1}I_{B2}I_{B3}I_{B4}w_{4}(t)=I_{IN}(t)$$

(32b) $$2nC_{2}U_{T}{\dot{w}}_{2}(t)+I_{B2}I_{B3}I_{B4}w_{4}(t)-\frac{I_{A1}}{I_{A0}I_{B2}}I_{IN}(t)=w_{1}(t)$$

(32c) $$2nC_{3}U_{T}{\dot{w}}_{3}(t)+I_{B3}I_{B4}w_{4}(t)-\frac{I_{A2}}{I_{A0}I_{B2}I_{B3}}I_{IN}(t)=w_{2}(t)$$

(32d) $$2nC_{4}U_{T}{\dot{w}}_{4}(t)+I_{B4}w_{4}(t)-\frac{I_{A3}}{I_{A0}I_{B2}I_{B3}I_{B4}}I_{IN}(t)=w_{3}(t).$$

This system of equations combined with *I_OUT_*(*t*) = *I*_*A*0_*I*_*B*2_*I*_*B*3_*I*_*B*4_*w*_4_(*t*) (which corresponds to the Bernoulli “*backbone*” TL loop *M_IN_M*_01_*M*_1_*M*_02_*M*_2_*M*_03_*M*_3_*M*_04_*M*_4_*M_OUT_*) results in the following transfer function *I_OUT_*(*s*)/*I_IN_*(*s*):

(33) $$\begin{array}{l} \text{​​​​}\frac{I_{OUT}(s)}{I_{IN}(s)}=\frac{I_{A3}}{2nC_{4}U_{T}}\text{ ​​}\\ \text{​​}\frac{s^{3}+\frac{I_{A2}I_{B4}}{I_{A3}2nC_{3}U_{T}}s^{2}+\frac{I_{A1}I_{B3}I_{B4}}{I_{A3}C_{2}C_{3}{\left(2nU_{T}\right)}^{2}}s+\frac{I_{A0}I_{B2}I_{B3}I_{B4}}{I_{A3}C_{1}C_{2}C_{3}{\left(2nU_{T}\right)}^{3}}}{\begin{array}{c} s^{4}+\frac{I_{B4}}{C_{4}2nU_{T}}s^{3}+\frac{I_{B3}I_{B4}}{C_{3}C_{4}{\left(2nU_{T}\right)}^{2}}s^{2}+\frac{I_{B2}I_{B3}I_{B4}}{C_{2}C_{3}C_{4}{\left(2nU_{T}\right)}^{3}}s\\ \;\;\;\;\;\;+\frac{I_{B1}I_{B2}I_{B3}I_{B4}}{C_{1}C_{2}C_{3}C_{4}{\left(2nU_{T}\right)}^{4}} \end{array}}. \end{array}$$

Clearly this BC-based analysis of log-domain structures with a large number of Bernoulli Cells seems to be simpler in its application, faster in its execution and less prone to errors for hand-analysis purposes.

## 6. General class of log-domain synaptic circuits

The systematic properties of the BCF emerge naturally from the analysis of all the previous synaptic circuit examples so far. The output currents of each circuit (*I_Syn_j__*(*t*)) were described either by a linear or a Bernoulli DE. However, all of them stem from the Bernoulli DE characterizing the BC-operator. The presence of the BC-operator in the aforementioned circuits allowed us to articulate certain “*rules-of-thumb*” regarding the analysis strategy that needs to be followed, when this category of circuits is investigated. These “*rules-of-thumb*” are only aiming to help the designer simplify the analysis/synthesis process, by exploiting the systematic nature of the BCF.

One may also note that for each one of the presented circuit topologies, a certain number of specific steps has been followed, in order to reach a final form of ODE that could describe the *I_Syn_j__*(*t*) current. Many of these steps served the purpose of clarifying to the reader that the BC-based analysis was behind the final form of the solution of the various output synaptic currents. Now that this point has been proved, it is time to group synaptic circuits under one general class of neuromorphic log-domain circuits, whose state-variable current could be governed by a specific set of equations, as shown in Table 1.

**Table 1:** **Forms of ODEs and their solutions stemming from the proposed general log-domain class of synaptic circuits**.

	**Linear equation**	**Bernoulli equation**
Form of ODE	*g*(*t*)*y*′_*t*_ = *f*_1_(*t*)*y* + *f*_0_(*t*)	*g*(*t*)*y*′_*t*_ = *f*_1_(*t*)*y* + *f_n_*(*t*)*y^n^*, *n* ≠ 0, 1
General solution	*y* = *Ke*^Λ^ + *e*^Λ^ ∫ *e*^−Λ^ *f*_0_(*t*)/*g*(*t*)*dt*	*y*^(1-*n*)^ = *Ke*^Λ^ + (1-*n*)*e*^Λ^ ∫ *e*^−Λ^ *f_n_*(*t*)/*g*(*t*)*dt*
Λ	∫*f*_1_(*t*)/*g*(*t*)*dt*	(1 − *n*) ∫ *f*_1_(*t*)/*g*(*t*)*dt*

**Figure 6:**
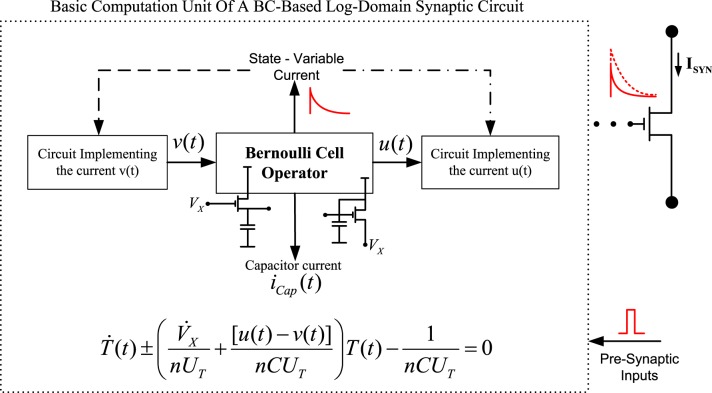
**Conceptual diagram describing the basic log-domain computation unit when a BC-operator is present**.

Regardless of the circuit topology that has been selected from the designer to implement a synaptic function, the BC-operator is always governed by the Bernoulli differential equation, whose linearised form is shown in (34) in general form. When a source-connected capacitor topology is present, (+) holds, while (−) holds when a diode-connected capacitor topology exists. As graphically shown in Figure 6, one can identify the dynamics of each circuit by simply examining the current relation that takes place in the circuit's “*basic computation unit*,” i.e. the BC. The parameter $\dot{V}$_*X*_ in (34) denotes the potential of either the source or the gate terminal of the BC MOST, depending on the type of the BC operator.

(34) $$\dot{T}(t)\pm (\frac{{\dot{V}}_{X}(t)}{nU_{T}}+\overset{\begin{array}{c} \text{Time Constant}\\ \text{Factors} \end{array}}{\overset{︷}{\frac{\left[u(t)-v(t)\right]}{nCU_{T}}}})T(t)-\frac{1}{nCU_{T}}=0.$$

The input/output currents *u*(*t*) and *v*(*t*) entering the BC (including the state-variable current *I_D_*(*t*) for the MOST case) are responsible for the charging and discharging phases of the circuit's capacitor; therefore, define the circuit's “*rate constants*” and consequently the form of the synaptic current. By identifying and analysing the BC-operator of each circuit, one is able to instantly define the dynamics of the circuit's output current by simply observing the relation between the BC's state variable current and the desired output current. A linear relation between the BC-state variable and the output synaptic current, e.g., *I_D_* ∝ constant × *I_Syn_* will lead to a Bernoulli ODE for the description of *I_Syn_*(*t*), while a non-linear relation, e.g., *I_D_* ∝ constant / *I_Syn_*, will lead to a linear ODE for the computation of *I_Syn_*(*t*) dynamics. All the above practical guidelines can be easily summarized into the following three basic circuit analysis steps/guidelines:

**Step 1:** Identify the BC-operator(s) by simply observing the connection between the circuit's capacitor(s) and the neighboring transistor(s);
**Step 2:** Once the BC-operator(s) is/are located, identify the relation between the state-variable current(s) of the operator(s) and the circuit's output current(s);
**Step 3:** If the relation between the BC operator(s) and the circuit's output current(s) is linear, then substitute the new relation for the output synaptic current *I_Syn_j__* = 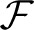
(*state variable current*) into (34) and solve the resulting differential equation. If their relation is of the form *I_D_* ∝ constant/*I_Syn_*, then a linear DE will be inevitably generated and the factor (*I_Syn_j__*/constant) should directly substitute the factor *T*(*t*) in (34);

The interesting attempt of Mitra et al. (2010) to provide a global parametric control of synaptic time constants and gain generates the ideal breeding ground for the application of this general class of synaptic dynamics created by the GBCF. For the various log-domain integrator circuit cases presented and analyzed in Mitra et al. (2010) (with a method similar to the one presented by Perry and Roberts in Perry and Roberts (1995), but “*silicon-synapse-oriented*”), the BC-operator will produce the exact similar solutions for the synaptic output current but all based on the different forms of the parameters *V_X_* and [*u*(*t*) − *v*(*t*)] sourcing from the different topologies. Table 2 provides an indicative number of neuromorphic topologies that could be easily implemented by the proposed GBCF.

**Table 2:** **An indicative list of neuromorphic circuits that could be described by the BC formalism**.

**Authors**	**Number of BCs**
Arthur and Boahen (2011)	3
Benjamin et al. (2012)	2
Boahen (1997)	1
Gao et al. (2012)	2
Hahnloser et al. (2000)	1
Hynna and Boahen (2003)	2
Merolla and Boahen (2006)	2
Mitra et al. (2010)	3
Thanapitak and Toumazou (2013)	1
van Schaik et al. (2010)	2
Wang and Liu (2010)	3
Yu and Cauwenberghs (2010)	1

At this point, an inverse question that arises is how one can design a synaptic aVLSI circuit, based on the fact that it will always be described by the specific type of equations? The answer to this synthesis question relates to the determination of the function *F* which links the BC state variable with *I_Syn_j__*(*t*) = 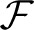
(*state variable current*) in such a way that the current *I_Syn_j__*(*t*) has certain pre-specified time profile properties. Issues, such as the practicability of the circuit, in conjunction with the form of the desired dynamics and its total chip area will definitely play a major role in the selection of the final form of the synaptic circuit. However, its “*analog heart*” implemented by the BC will be identical in all cases.

Finally, for the sake of completeness, it would be useful to remind to the reader that all previous mathematical formulas have been derived based on the valid assumption that the voltage difference between the bulk and source terminal of the subthreshold MOSTs is zero, i.e. *V_BS_* = 0. However, this assumption represents the ideal operation of a weakly-inverted MOST, without taking into consideration the impact of the “*body effect*” upon the devices' overall performance. Other indicative limitations that restrain a MOST in the subthreshold regime and affect the device's performance are the output resistance, matching, bandwidth and noise limitations, as well as short-channel effects, such as the drain induced barrier lowering (DIBL) (Andreou and Boahen, 1996). Useful mathematical relationships that manage to quantify the aforementioned limitations and therefore, provide useful guidelines when it comes to the selection of critical MOST parameters can be found in Andreou and Boahen (1996).

The most common limitation for a MOST in weak-inversion involves the non-zero voltage difference between its bulk and source terminals. The effect of this limitation upon the GBCF can be shown in the following indicative mathematical analysis. Starting from the full mathematical expression that defines the current of a subthreshold n-type MOST (Tsividis, 1996), assuming again that the device is in deep saturation, it holds that:

$$I_{D}=\frac{W}{L}I_{D_{O}}\;exp\left(\frac{(n-1)V_{BS}}{nU_{T}}\right)\;exp\left(\frac{V_{GS}-V_{TH}}{nU_{T}}\right),$$

where *I_D_O__* is a process-dependent parameter, *W*/*L* is the aspect ratio of the transistor and *n* is again the subthreshold slope parameter (Tsividis, 1996). This expression can be re-written equivalently as:

$$I_{D}={\overset{´}{I}}_{D_{O}}\;exp\left(\frac{V_{GS}+(n-1)V_{BS}}{nU_{T}}\right),$$

where Í*_D_O__* = (*W*/*L*) *I_D_O__ exp*(−*V_TH_*/(*nU_T_*)). For the circuit topologies originally mentioned in Section 2 and under the assumption that the bulk terminal of the device has been tied to a constant voltage source, the time derivative of the new expression of *I_D_* current, when a linear capacitor is connected to its source terminal would lead to the following linearised expression (using the same transformation as shown in section 2, i.e. *I_D_*(*t*) = 1/*T*(*t*)), depending on the type of the BC operator:

(35) $$\dot{T}(t)\pm (\frac{{\dot{V}}_{X}(t)}{nU_{T}}+\overset{\begin{array}{c} \text{Time Constant}\\ \text{Factors} \end{array}}{\overset{︷}{\frac{\left[u(t)-v(t)\right]}{CU_{T}}}})T(t)-\frac{1}{CU_{T}}=0,$$

with the parameter $\dot{V}$_*X*_ in (35) denoting again the potential of either the source or the gate terminal of the BC MOST, depending on the type of the BC operator. The interested reader should verify that unit consistency has been preserved in (35), in complete analogy with (34). Interestingly enough, from (35), it can be extracted that the time constant factor does not exhibit a dependence upon the subthreshold slope parameter *n*. In other words, the effect of this MOST non-ideality has led to the following conclusion regarding GBCF, i.e.:

$${\begin{array}{c} \text{Silicon Synaptic}\\ \text{ Time Constant Factor} \end{array}}_{V_{BS}\;\neq \;0}\text{​}=\text{​ }n\times {\begin{array}{c} \text{Silicon Synaptic}\\ \text{ Time Constant Factor} \end{array}}_{V_{BS}\;=\;0}.$$

Once again, in complete analogy with the above analysis, the interested reader could investigate the effect of other MOST limitations upon the overall performance and consequently acquire handy relations that could inform, in a quantitatively and qualitative manner, about the deviation of the device from its ideal behavior.

## 7. Discussion

The paper discussed in a tutorial manner an alternative transistor-level method to treat log-domain synaptic circuits. An extended version of the BCF proved the existence of BC-operators not only when a linear capacitor is connected to the emitter/source of a transistor but also when a linear capacitor is connected to the base/gate of a diode-connected transistor. The usefulness of this endeavor lies in the handiness of the BCF when it comes to the analysis (or synthesis) of linear and/or non-linear log-domain circuits. By providing one more topology, the “*diode-connected*” BC operator where the BCF applies, this paper extends the solid mathematical background, where engineers can rely upon when it comes to the study and design of log-domain circuits for neuromorphic or other applications.

The analysis of the synaptic circuits presented and analyzed in the previous sections stresses the taxonomic prowess of the BCF. The core operation of synaptic circuits is based ultimately on the exponentiation of a capacitor voltage during its charging/discharging phases facilitated by a MOST. The rest of the circuit is used to provide the correct weights and time constants of the artificial synapse, so that a more faithful representation of the biological synapse model is achieved. The independent nature of the BC-operator's input and output currents [*u*(*t*) and *v*(*t*)] allows, in principle, for the designer to determine the appropriate circuit topology that will generate the desired dynamics.

It is left to the readers to evaluate the benefits of using the aforementioned parsimonious formalism for log-domain synaptic and other neuromorphic circuits. It is genuinely hoped that the tutorial nature of this paper will provide a helping hand to engineers wishing to explore aVLSI synaptic circuits in a more intuitive way, streamlining their mathematical analysis in a rigorous manner.

### Conflict of interest statement

The authors declare that the research was conducted in the absence of any commercial or financial relationships that could be construed as a potential conflict of interest.

## Footnotes

^1^The analysis method articulated in Mulder et al. (1997), Mulder (1998) concerned BJT log-domain topologies. Here it is applied for MOSTs log-domain topologies.

## Appendix

Starting for the fact that in the given circuit *I_Syn_* ≫ *I_Gain_*, then their full expressions should be:

$$I_{O}\;e^{\frac{V_{DD}-V_{Syn}}{nU_{T}}}\gg I_{O}\;e^{\frac{V_{DD}-V_{THR}}{nU_{T}}}$$

or

$$e^{-\frac{V_{Syn}}{nU_{T}}}\gg e^{-\frac{V_{THR}}{nU_{T}}}$$

which easily leads to the following inequality for the two voltages: *V_THR_* ≫ *V_Syn_*, or equivalently *I_THR_* ≫ *I_IN_*.
